# Feasibility and Safety of Endoluminal Radiofrequency Ablation as a Rescue Treatment for Bilateral Metal Stent Obstruction Due to Tumor Ingrowth in the Hilum: A Pilot Study

**DOI:** 10.3390/jcm10050952

**Published:** 2021-03-01

**Authors:** Hoonsub So, Chi Hyuk Oh, Tae Jun Song, Hyun Woo Lee, Jun Seong Hwang, Sung Woo Ko, Dongwook Oh, Do Hyun Park, Sang Soo Lee, Dong-Wan Seo, Sung Koo Lee, Myung-Hwan Kim

**Affiliations:** 1 Department of Internal Medicine, Ulsan University Hospital, University of Ulsan College of Medicine, Ulsan 44033, Korea; hoon3112@gmail.com; 2 Division of Gastroenterology and Hepatology, Department of Internal Medicine, Kyung Hee University Hospital, Seoul 02453, Korea; harrison@daum.net; 3 Asan Medical Center, Division of Gastroenterology, Department of Internal Medicine, University of Ulsan College of Medicine, Seoul 05505, Korea; dongwook.oh1@gmail.com (D.O.); dhpark@amc.seoul.kr (D.H.P.); ssleedr@amc.seoul.kr (S.S.L.); dwseoamc@amc.seoul.kr (D.-W.S.); sklee@amc.seoul.kr (S.K.L.); mhkim@amc.seoul.kr (M.-H.K.); 4 Division of Gastroenterology, Department of Internal Medicine, Gimpo Woori Hospital, Gimpo 10099, Korea; im_lhw@naver.com; 5 Department of Internal Medicine, Inje University College of Medicine, Haeundae Paik Hospital, Busan 48108, Korea; hjs_snu@naver.com; 6 Department of Internal Medicine, Eunpyeong St. Mary’s Hospital, Catholic University, Seoul 03312, Korea; gogo930@catholic.ac.kr

**Keywords:** cholangiocarcinoma, endoscopic retrograde cholangiopancreatography, radiofrequency ablation

## Abstract

**Background.** Radiofrequency ablation (RFA) is a palliative method known for its application in the endoscopic treatment of malignant bile duct obstruction. It may be a useful rescue method for metal stent malfunction caused by tumor ingrowth. This study aimed to examine the feasibility and safety of endoluminal RFA for occluded bilateral hilar metal stents due to tumor ingrowth in patients with malignant hilar bile duct obstruction. **Methods:** From March 2016 to June 2018, 11 patients with unresectable malignant hilar bile duct stricture with occluded bilateral hilar metal stents due to tumor ingrowth were enrolled. Endoluminal RFA was performed through a novel temperature-controlled catheter at a setting of 7 W power for 120 s with a target temperature of 80 °C via endoscopic retrograde cholangiopancreatography (ERCP). The patients’ demographics, clinical outcomes, and adverse events were investigated. **Results:** The median age was 64 (interquartile range, 54–72) years. All RFA procedures were successful. Clinical success was achieved in eight patients (72.7%). During the follow-up, eight patients (72.7%) showed stent dysfunction, and the median patency after RFA was 50 days (95% confidence interval (CI): 34–not available (NA)). All stent dysfunctions were successfully managed with ERCP. Ten patients died, and the median overall survival was 289 days (95% CI, 107–NA) from RFA to death. There was one case of mild abdominal pain after the procedure without serious adverse events. **Conclusions:** As a rescue therapy for occluded bilateral hilar metal stents due to tumor ingrowth, endoluminal RFA seemed to be safe and useful in selected patients.

## 1. Introduction

Resolution of biliary obstruction is the most important factor for the survival and quality of life of patients with malignant biliary obstruction. Insertion of self-expandable metal stents (SEMSs) is the standard palliative treatment for unresectable malignant biliary obstruction in patients with a life expectancy of >3 months, as SEMSs have demonstrated longer patency than plastic stents for maintaining biliary drainage [1,2,3]. In the case of hilar obstruction, especially Bismuth type III or IV strictures, bilateral metal stenting is recommend, as some studies have shown longer survival, better patency, and fewer reinterventions with the use of bilateral metal stents [4]. However, in the case of obstruction of a bilaterally inserted stent by tumor ingrowth, endoscopic revision is technically challenging because of the small space, complex anatomy, and disturbing mesh. The current treatment options for an occluded bilateral SEMS include insertion of a percutaneous external drain or secondary stenting with or without preceding balloon dilatation through the occluded primary stent [5]. Therefore, there is a growing need for local control of obstructed bilateral metal stents, and radiofrequency ablation (RFA) has been one of the candidates for resolving this problem.

RFA is a well-established ablative procedure that causes coagulative necrosis, leading to local destruction of target tissue in many solid-organ malignancies [6]. Novel RFA catheters have opened the possibility of new treatments and have been successfully used with percutaneous and endoscopic approaches for palliative intraductal ablation in patients with malignant bile duct obstruction. Intraductal RFA combined with biliary SEMS also seems to be safe and to lead to improved stent patency and prolonged survival [7,8,9]. More recently, there has been increasing interest in the role of RFA in inducing localized necrosis of tumor ingrowth or overgrowth to increase the patency of occluded biliary metal stents in patients with malignant biliary obstruction. The initial experience in clearing occluded stents has demonstrated the safety and efficacy of RFA. However, RFA remains limited by its percutaneous approach and its application to mostly extrahepatic biliary obstruction [10,11]. As hilar lesions are close to the hepatic artery and portal vein, there have been concerns about injury of the vessels around the bile duct (which can result in massive hemobilia), hepatic artery pseudoaneurysm, or hepatic infarction. There has been no definite report on endoluminal RFA through an endoscopic approach for occluded metal stents in patients with malignant hilar bile duct obstruction.

The aim of this pilot study was to evaluate the feasibility and safety of endoluminal RFA with automatic temperature control through the endoscopic approach for occluded bilateral hilar biliary metal stents in patients with unresectable hilar bile duct obstruction.

## 2. Methods

### Patients

Patients were enrolled in this study from March 2016 to June 2018. The inclusion criteria were as follows: (i) biopsy-proven unresectable hilar cholangiocarcinoma, (ii) previous bilateral metal stent insertion with the stent-in-stent method, and (iii) stent malfunction due to tumor ingrowth. Before the RFA treatment, SEMS malfunction due to tumor ingrowth was identified with magnetic resonance cholangiopancreatography (MRCP) or endoscopic retrograde cholangiopancreatography (ERCP). The demographics, technical success, clinical success, symptom-free survival, overall survival, and procedure-related adverse events were investigated in all patients. All patients had been prospectively included, and the data were retrospectively analyzed in this study. This study was approved by the institutional review board of Asan Medical Center (number 2016-1059), and written informed consent was obtained from all patients.

## 3. Definitions

Imaging examinations (computed tomography (CT), MRCP, and ERCP) for the diagnosis of stent occlusion were performed when a patient presented with cholangitis or elevation of the serum bilirubin level [12]. Tumor ingrowth was also diagnosed when cholangiography showed a stricture within the stent, with a similar appearance to that of the original malignant stricture. Technical success of endoluminal RFA was defined as the positioning of the RFA catheter at the stricture site and achieving ablation as intended [13]. Clinical success was defined as a normalization of bilirubin level or a decrease in bilirubin level to >50% of the pretreatment value within the first month without recurrent cholangitis or biliary sepsis [14]. Stent patency was defined as the time from RFA to the next obstructive symptom or sign of biliary obstruction. Overall survival was calculated from the time of RFA to the last day of follow-up or death. Adverse events were defined and classified into intra-procedure, post-procedure (up to 14 days), or late events, based on a lexicon for endoscopic adverse events [15].

### 3.1. RFA Catheter and Power Settings

**Figure 1:**
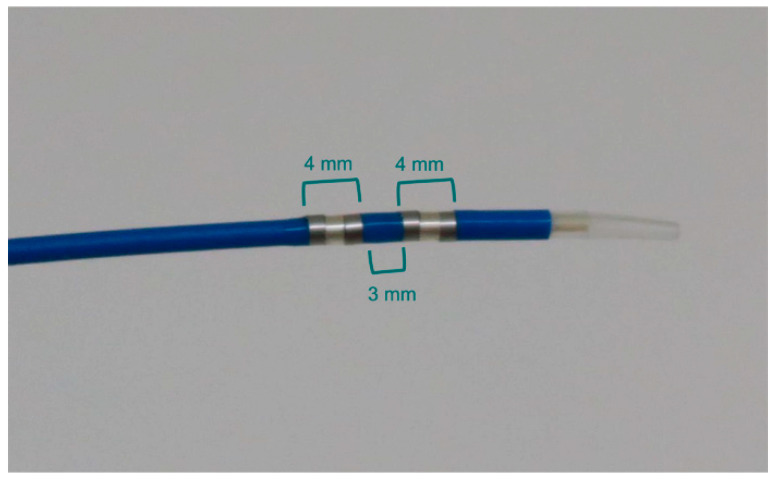
Electrode catheter for endoluminal radiofrequency ablation (RFA). The distal end of the catheter has two circumferential 4-mm-wide RFA electrodes, separated by a distance of 3 mm, providing cylindrical ablation over an 11-mm length.

RFA was performed with an endoluminal RFA electrode (ELRA; STARmed, Goyang, Korea). The probe used is a flexible, 7-Fr, bipolar device with two 4-mm ring electrodes (each 3 mm apart), and the endoscopic approach was used for the cylindrical ablation of tissue over a length of 1.1 cm (Figure 1). If the probe contacted a metal stent, the power was automatically stopped by switching from bipolar to unipolar mode. In this condition, the probe position was adjusted to allow for continuation of the ablation. The probe contains a temperature sensor inside the ablation active zone, which prevents overheating. It is compatible with standard side-viewing endoscopes (TJF-260; Olympus, Tokyo, Japan), with a working channel length of 175 cm. Ablation was achieved using an RFA generator (VIVA combo; STARmed, Goyang, Korea). This generator automatically turns on and off according to the specific presetting of the electrode temperature to avoid excessive heating during the RFA process. The preset target temperature was 80 °C.

### 3.2. Endoluminal RFA Procedure

**Figure 2:**
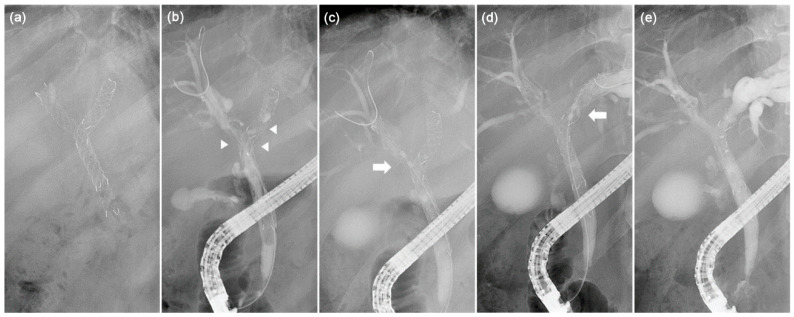
Application of endoscopic intraductal radiofrequency ablation (RFA) in a 64-year-old patient with hilar cholangiocarcinoma. (**a**) Fluoroscopic view showing previous bilateral metal stents placed using the stent-in-stent method. (**b**) Cholangiogram showing partial occlusion of the metallic stent in the hilum and total occlusion in the left hepatic duct (arrowhead) due to tumor ingrowth. (**c**,**d**) RFA catheter (arrow) inserted in the hepatic duct and ablation performed within the metal stent. (**e**) Cholangiogram showing restoration of luminal strictures after RFA.

After the selective cannulation of the common bile duct followed by acquisition of a cholangiogram to delineate the stricture, the RFA probe was placed over the guidewire at the area of the biliary stricture within the SEMS under fluoroscopic guidance. If both sides were obstructed by tumor ingrowth, both were ablated. Ablation was performed with an RFA generator at the desired setting, which delivered a radiofrequency energy of 7 W for 120 s per lesion. This energy setting was chosen to minimize unexpected adverse events, based on a previous study [16]. The probe with the shortest length (1.1 cm) was used to minimize contact with the wire of the SEMS. RFA was repeatedly applied to ensure ablation of the whole length of the stricture, with adjustments to avoid overlapping. After the completion of RFA, a temporary endoscopic nasobiliary drainage catheter or 7-Fr plastic stents were placed, according to the endoscopist’s discretion, to prevent cholangitis (Figure 2).

### 3.3. Post-Intervention Follow-Up

The patients underwent plain abdominal radiography and blood tests for complete blood count, liver function, and pancreatic enzyme assessments in order to evaluate the adverse events of the procedure on the next day after RFA. Thereafter, all patients were regularly followed up 1 week later and then once a month with clinical examination and laboratory tests. CT scan was performed when clinical signs of biliary obstruction were observed, or cholangitis was suspected during the follow-up.

## 4. Results

### 4.1. Patient Demographics

A total of 11 patients were enrolled. The median age was 64 years (interquartile range, 54–72), and the male-to-female ratio was 6:5. All patients had a diagnosis of unresectable hilar cholangiocarcinoma (Bismuth classification type II, *n* = 3; type IIIa, *n* = 2; type IIIb, *n* = 2; type IV, *n* = 4) and had undergone insertion of bilateral uncovered metal stents with the stent-in-stent method (8/10 mm in diameter, 6/8/10 mm in length, Niti-S LCD biliary stent; Taewoong Medical, Seoul, Korea). The median time from the diagnosis of cancer to RFA was 245 days (interquartile range, 198–338 days). Nine patients had undergone systemic chemotherapy or chemoradiotherapy before RFA, and two had been receiving best supportive care. The patient demographics and clinical characteristics are shown in Table 1.

### 4.2. Outcomes of Temperature-Controlled Endoluminal RFA

**Figure 3:**
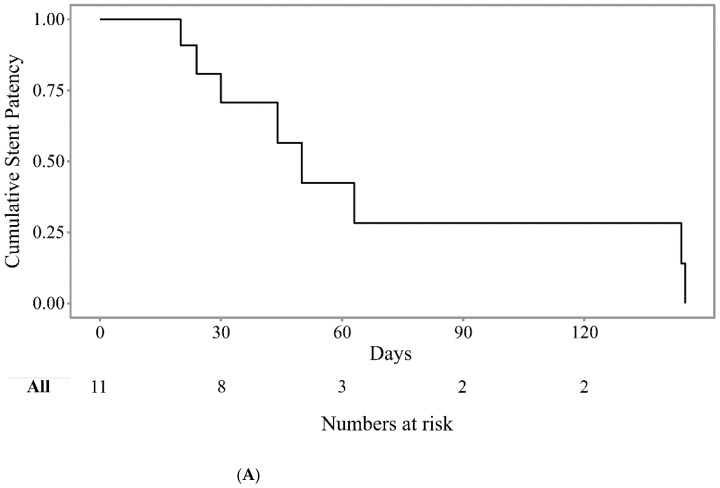

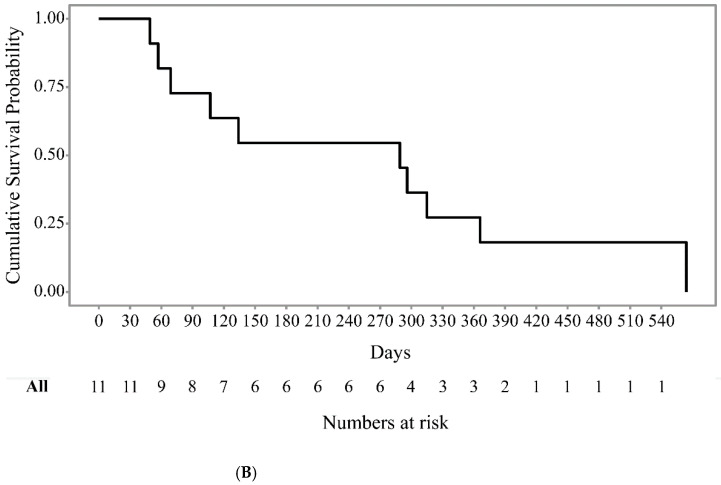
Kaplan–Meier curve showing the cumulative stent patency (**A**) and cumulative patient survival (**B**) after endobiliary radiofrequency ablation.

In this study, endoluminal RFA was successfully achieved inside the stent to reestablish the patency of the occluded SEMS without any technical problems in all patients. RFA was applied to both sides (*n* = 5), left (*n* = 2), right (*n* = 1), and common hepatic duct (*n* = 3) in one session Clinical success was achieved in eight patients (72.7%). Three patients had clinical failure by cholangitis (*n* = 2) and by advanced cancer (*n* = 1). Two with cholangitis were managed with reintervention by ERCP within 1 month and one with best supportive care. Stent dysfunction after RFA occurred in eight patients (72.7%) during the follow-up periods, and the median stent patency after the RFA procedure was 50 days (95% confidence interval (CI): 34–not available (NA)) (Figure 3A). The overall survival was 289 days (95% CI, 107–NA) after the RFA procedures (Figure 3B). There was no 30-day mortality after RFA. In terms of adverse events, one patient developed abdominal pain that improved with analgesics. There were no procedure-related mortality and other adverse events, such as bleeding, bile duct perforation, and hepatic infarction. The treatment details are summarized in Table 2.

## 5. Discussion

This is the first report on the feasibility and safety of temperature-controlled endoluminal RFA in patients with hilar cholangiocarcinoma with occluded bilateral hilar SEMS. The technical success rate was 100%, without significant adverse events. The clinical success rate was 72.7%, with a median stent patency after the RFA procedure of 50 days. Our experience showed that temperature-controlled endoluminal RFA can be safely applied for the rescue of obstructed bilateral SEMSs in the hilar area. When SEMSs placed bilaterally for hilar cholangiocarcinoma are occluded by tumor ingrowth, revision is complex and challenging. Percutaneous transhepatic biliary drainage (PTBD), ERCP with placement of additional plastic stents, or endoscopic ultrasound (EUS)-guided drainage can be attempted as a rescue procedure. However, PTBD has a relatively high rate of complications and causes discomfort in patients related to external drainage. Additional stent placement through previously inserted bilateral metal stents using ERCP is technically complex and challenging. EUS-guided drainage is not always feasible in every patient. Therefore, local ablative therapy can be attempted as a rescue therapy, and the aim of this study was to evaluate the feasibility and safety of endoluminal RFA as a rescue method for those cases.

RFA is one of the local ablative techniques that can be performed in the bile duct during ERCP. Tumor ablation with heat using a novel bipolar probe has been reported to be feasible and technically simple to perform. Several reports on RFA with the endoscopic and percutaneous transhepatic approach for malignant biliary obstruction have shown its potential role in delaying tumor growth and prolonging stent patency [17,18]. A recent meta-analysis demonstrated that biliary RFA had the advantages of prolonging stent patency (mean difference, 50.6 days; 95% CI, 32.83–68.48) and improving patient survival (hazard ratio, 1.395; 95% CI, 1.145–0.7; *p* < 0.001) without causing serious adverse events [9]. RFA may play a role as a rescue therapy for stent obstruction by tumor ingrowth; however, its efficacy and safety still lack evidence [18,19,20,21].

Theoretically, endoluminal RFA may also have the effect of reducing the tumor volume and inducing systemic anti-tumor response [9]. RFA is reported to stimulate tumor-specific cytotoxic T-cell activity by inducing necrotic cell death [22,23]. A mouse model demonstrated a weak, detectable immune response against tumor cells after RFA [24]. A study on RFA combined with surgical resection for non-small cell lung cancer showed intense infiltrations of CD4+ and CD8+ lymphocytes at the perimeter of the RFA-treated tumor tissue. Proinflammatory, immunostimulatory IFNγ-secreting, and immunostimulatory BDCA3þ/B7-H3- dendritic cells were increased in blood test after RFA [25]. Not only the ablation itself but also immune response may contribute to better survival or longer patency; future studies will be required to investigate the contributions of each of these factors. The major concern about biliary endoluminal RFA is thermal injury to surrounding structures, including the intact bile duct, hepatic artery, portal vein, cystic duct, liver, and pancreas, which might lead to bile duct perforation, hemobilia, hepatic infarction, cholecystitis, or pancreatitis [26]. In particular, the perihilar bile duct wall is thinner than the extrahepatic bile duct, and branches of the hepatic artery may run within 1 mm of the proximal common hepatic duct [27]. Therefore, the hilar bile duct may be more vulnerable to the development of serious vascular adverse events, including massive hemobilia, pseudoaneurysm, or hepatic infarction due to arterial thrombosis, after RFA. Kadayifci et al. showed the feasibility of RFA for occluded stent ahead of our study. Among 25 patients enrolled, 14 (56%) were successful with ablation, and the patency after ablation was significantly longer compared to the additional plastic stent group without RFA (119.5 vs. 65.3 days, *p* = 0.03) [11]. However, most of the dominant strictures in the study were in the lower or mid-third of common bile duct. Yoon et al. showed in an in vitro experiment that RFA is safe in the presence of a metal stent. When RFA (10 W, temperature range 65–75 °C, 30 s) was applied within the uncovered metal stent, coagulation was confined to the electrodes. The mechanism was explained as follows: as the coagulated area expanded and contacted the wire of the uncovered metal stent, the impedance between the electrodes was rapidly decreased by the current flow to the wire. In a porcine bile duct model, necrosis after RFA was markedly reduced compared with that in the unstented bile duct and was limited to the superficial mucosa with no apparent damage to the metal stent [28]. On the basis of this experiment, the stent may provide a protective effect to surrounding structures against thermal injury. This is in line with our study results in which no major adverse events were observed, which means that, theoretically, RFA can be suitably indicated. Furthermore, we chose a short probe with temperature-controlling function for safety. Therefore, endobiliary RFA can safely ablate an ingrowing tumor inside the stent.

There is yet no standardized optimal heating energy dose and ablation time in endoluminal RFA for bile duct cancer. Other studies mostly used 7–10 W and 60–120 s [29]. We used a setting of 7 W and 120 s with an 11-mm probe. In a study using the ELRA probe, the median ablation depths were 2.7 mm (range 2.5–4.3 mm) using 10 W power with a 33-mm catheter for 120 s and 2.1 mm (range 1.7–2.4 mm) using 7 W power with an 18-mm catheter [16]. A pathologic evaluation after endobiliary RFA for distal extrahepatic cholangiocarcinoma reported that the median effective ablation length (histological ablation length/fluoroscopic ablation length) was 72.0% (range, 42.1–95.3%) with a median maximal ablation depth of 4.0 mm (range, 1–6 mm) [14]. However, it is technically challenging to use a longer probe with higher power (10 W) in the hilar area, owing to the complex anatomy. Moreover, a longer probe can easily touch the mesh of the stent, thus leading to early termination of RFA. Therefore, we suggest that, for better efficacy, repeated RFA with a probe with a short length while covering enough range beyond the actual stricture may be recommended.

This pilot study has limitations related to its retrospective nature. Additionally, our study included a small number of patients in a single arm.

In conclusion, biliary endoluminal RFA may be a therapeutic alternative for occluded bilateral SEMSs in patients with malignant hilar bile duct obstruction. This preliminary study may serve as a basis for larger trials and multidisciplinary studies assessing the use of endoluminal RFA for occluded bilateral hilar SEMSs due to tumor ingrowth as a potential therapy for selected patients with advanced bile duct cancer that cannot be treated with surgery.

## Figures and Tables

**Table 1:** Baseline characteristics of the included patients.

Patient-Related Characteristics	Value
Median age, y	64 (54–72)
Male:female	6:5
Cholangiocarcinoma ^a^, *n* (%)	
Type II	3 (27)
Type IIIa	2 (18)
Type IIIb	2 (18)
Type IV	4 (37)
Median time from diagnosis to RFA, days	245 (198–338.0)

All values are presented as median (interquartile range) and number (%). RFA: radiofrequency ablation. ^a^ Type was classified according to the Bismuth–Corlette classification of perihilar cholangiocarcinoma.

**Table 2:** Outcomes and adverse events of endobiliary radiofrequency ablation for hilar cholangiocarcinoma.

Outcomes	*n* = 11
Technical success, *n* (%)	11(100)
Clinical success, *n* (%)	8 (72.7)
Stent dysfunction, *n* (%)	8 (72.7)
Stent patency, days	50 (95% CI, 34–NA)
Death, *n* (%)	10 (90.9)
Overall survival, days	289 (95% CI, 107–NA)
Adverse events	
Intra-procedure	None
Post-procedure, *n*	
Abdominal pain	1

CI: confidence interval; NA: not available.

## Data Availability

The data presented in this study are available on request from the corresponding author.
